# Pharmacological treatments in ARDS; a state-of-the-art update

**DOI:** 10.1186/1741-7015-11-166

**Published:** 2013-08-20

**Authors:** Andrew James Boyle, Rob Mac Sweeney, Daniel Francis McAuley

**Affiliations:** 1 Centre for Infection and Immunity, Health Sciences Building, Queen’s University Belfast, 97 Lisburn Road, Belfast BT9 7BL, UK; 2 Regional Intensive Care Unit, Royal Victoria Hospital, 274 Grosvenor Road, Belfast BT12 6BA, UK

**Keywords:** Acute lung injury, Acute respiratory distress syndrome

## Abstract

Despite its high incidence and devastating outcomes, acute respiratory distress syndrome (ARDS) has no specific treatment, with effective therapy currently limited to minimizing potentially harmful ventilation and avoiding a positive fluid balance. Many pharmacological therapies have been investigated with limited success to date. In this review article we provide a state-of-the-art update on recent and ongoing trials, as well as reviewing promising future pharmacological therapies in ARDS.

## Introduction

Despite its high incidence and devastating outcomes [1,2], acute respiratory distress syndrome (ARDS) has no specific treatment, with effective therapy currently limited to minimizing potentially harmful ventilation and avoiding a positive fluid balance. ARDS is characterized by breakdown of the alveolar-capillary barrier, leading to flooding of the alveolar space producing the classical chest radiograph of bilateral pulmonary infiltrates. This non-cardiogenic pulmonary edema is associated with impaired oxygenation, as measured by the PaO_2_/FiO_2_ (P/F) ratio, with a lower P/F ratio indicating more severe hypoxia. Acute lung injury (ALI) is defined as a P/F ratio <300 mmHg (40kPa) and ARDS is a sub-group defined on the basis of more severely impaired oxygenation with a P/F ratio <200 mmHg (26.7 kPa).

Since it was first described in 1967 [3], and despite over 40 years of research, few pharmacological therapies have emerged for ARDS. We limited the search strategy for this state-of-the-art update review to recent randomized controlled trials and meta-analyses, as well as a review of promising potential future pharmacological therapies in ARDS in an adult setting.

### Neuromuscular blockade

Lung protective ventilation can be achieved in the majority of patients without using neuromuscular blockade (NMB) [4]; however, initial small studies eliminating patient effort via skeletal muscle inhibition with NMB improved patient-ventilator synchrony, as evidenced by reduced airway pressures and improved chest wall compliance. Therefore, in the severely hypoxemic ARDS patient, NMB may permit lower-pressure, lower-tidal volume ventilation with a consequent reduction in ventilator-induced lung injury. These beneficial effects led to a multi-center, randomized, placebo-controlled trial to assess the effect of NMB upon mortality [5] (Table 1). This showed that infusion with cisatracuriumbesylate within 48 hours of mechanical ventilation in patients with moderate ARDS improved 90-day survival. However, no difference was noted between the intervention and placebo groups until Day 20. The biological mechanism by which NMB improves late but not early outcome is unclear. While promising, the protective effect of neuromuscular blockade needs to be confirmed in a further phase 3 trial.

**Table 1:** Characteristics of trials to date

**Study title and abbreviation**	**Design (all placebo-controlled)**	**Population of ALI/ARDS A) Timing from ALI onset B) P/F ratio**	**Number recruited**	**Intervention**	**Primary outcome**	**Result (intervention vs control)**	**Mortality (intervention vs control)**
**Neuromuscular Blockade in Early ARDS **[5]	Phase 2 RCT	A) 48 hours B) <150	340	Cisatracuriumbesylate: 15 mg initially, then 37.5 mg per hour for 48 hours	90-day survival	31.6% vs 40.7% (*P*= 0.08)	28-day: 23.7% vs 33.3% (*P*= 0.05)
**The β-Agonist Lung Injury Trial (BALTI) **[6]	Phase 1 RCT	A) 48 hours B) <300	40	Intravenous (IV) salbutamol for seven days (15 μg kg^-1^ h^-1^)	Extravascular lung water (EVLW) at Day 7	9.2 ± 6 vs 13.2 ±3 ml kg^-1^ (*P*= 0.04)	28-day: 58% vs 66% (*P*= 0.4)
**Randomized, Placebo-Controlled Clinical Trial of an Aerosolized β2-Agonist for Treatment of Acute Lung Injury (ALTA) **[7]	Phase 2 RCT	A) 48 hours B) < 300	282	Inhaled salbutamol (5 mg) every 4 hours for 10 days/24 hours after extubation	Ventilator-free days (VFD)	**Stopped early** 14.4 ± 0.9 vs 16.6 ± 0.9 (*P*= 0.087)	Death before discharge: 24.3 ± 3.5 vs 18.5 ± 3.4 (*P*= 0.261)
**Effect of Intravenous β-2 Agonist Treatment on Clinical Outcomes in Acute Respiratory Distress Syndrome (BALTI-2) **[8]	Phase 2 RCT	A) 72 hours B) <200	326	IV salbutamol for seven days (15 μg kg^-1^ (ideal body weight) h^-1^)	28-day mortality	**Stopped early** 34% vs 23% (*P*= 0.03)	
**Neutrophil Elastase Inhibition in Acute Lung Injury (STRIVE) **[9]	Phase 3 RCT	A) 48 hours B) <300	492	Sivelestat infusion	1. 28-day mortality2. VFD	**Stopped early** 1 26.6% vs 26% (*P*= 0.847)2. 11.4 ±10.27 vs 11.9 ± 10.1 (*P*= 0.536)	
**Efficacy and Safety of Corticosteroids for Persistent ARDS (LaSRS) **[14]	Phase 2 RCT	A) 7 to 28 days B) P/F <200	180	Moderate-dose IV methylprednisolone, for up to 25 days	60-day mortality	29.2% vs 28.6% (*P*= 1.0)	
**Methylprednisolone Infusion in Early Severe ARDS **[15]	Phase 1 RCT	A) 72 hours B) <300	91	Low-dose IV methylprednisolone, for up to 28 days	Improvement in Lung Injury Score by Day 7	69.8% vs 35.7% (*P*= 0.002)	Hospital survival 76.2% vs 57.1% (*P*= 0.07)
**A Randomized Clinical Trial of Hydroxymethylglutaryl–Coenzyme A Reductase Inhibition for Acute Lung Injury (HARP) **[19]	Phase 2 RCT	A) 48 hours B) <300	60	Simvastatin 80 mg daily, up to 14 days	Reduction in EVLW indexed to actual body weight	13.7 vs 13.4 (*P*= 0.90) Improvements in secondary outcomes	Hospital survival: 19 vs 19 (*P*= 1.0)
**Nebulized Heparin is Associated with Fewer Days of Mechanical Ventilation in Critically Ill Patients: a Randomized Controlled Trial **[21]	Phase 2 RCT	Patients expected to require ventilation for >48 hours, and within 24 hours of ventilation	50	Heparin 25,000 units every 4 to 6 hours, for up to 14 days	Average daily P/F ratio	194.2 ± 62.8 vs 187 ± 38.6 mmHg (*P*= 0.6) Improvements in secondary outcomes	28-day: 20% vs 16% (*P*= 0.7)

### β-adrenergic agonists

Alveolar edema is a central feature of ARDS, contributing to limitation of gaseous exchange and ventilatory failure. Experimental data suggest β-adrenergic agonists could accelerate alveolar fluid clearance, as well as provide cytoprotection, increased surfactant secretion and decreased endothelial permeability.

Based on the proposed effect upon alveolar fluid clearance, the β-Agonist Lung Injury Trial (BALTI) randomized 60 patients to IV salbutamol or placebo for seven days [6]. In this small, single center study, salbutamol therapy significantly reduced extravascular lung water at Day 7 compared with placebo (Table 1).

Subsequently, two large multi-center, randomized placebo-controlled trials were initiated. The first American study, ALTA (Albuterol Treatment for Acute Lung Injury) [7], enrolled 282 patients with ALI, but failed to demonstrate a difference in ventilator-free days between those receiving inhaled β-agonist therapy and those given placebo, and was stopped early as it breached the futility boundary (Table 1). In the most severely ill, as defined by the presence of shock at randomization, length of stay was significantly increased, suggesting worse outcome in this sub-group.

BALTI-2 was a concurrent UK multi-center study investigating intravenous salbutamol in patients with ARDS, but was terminated early due to excess mortality in the group receiving IV salbutamol [8] (Table 1).

On the basis of these larger trials, β-agonists should be avoided in patients with ALI. It is hypothesized that β-agonists may have a harmful cardiac effect, stimulating tachyarrhythmias and cardiac ischemia, resulting in a poorer outcome.

### Neutrophil elastase inhibitors

Neutrophil elastase (NE) is a serine protease found in neutrophil granules and has a range of physiological effects, including anti-microbial actions and modification of tissue repair and inflammation. Excessive NE is capable of degrading endothelial basement membrane, and has been implicated in the pathogenesis of ARDS. Following a positive study in Japan, silvelestat, a neutrophil elastase inhibitor, was investigated in an international randomized, double-blind, placebo-controlled, multi-center phase III trial (STRIVE) [9] (Table 1). The study was stopped prematurely due to an increase in 180-day all-cause mortality.

A more recent meta-analysis of eight clinical trials (including STRIVE) investigating silvelestat has shown it to have no effect on short-term mortality, and a worse outcome for 180-day mortality (Risk Ratio (RR) 1.27, CI 1.00 to 1.62) [10].

### Corticosteroids

Given their effective anti-inflammatory properties, there has been extensive interest in the potential role of corticosteroids in both the prevention and treatment of ARDS. Different regimens have been investigated, varying from short courses of high-dose steroids to prolonged courses of lower doses.

High dose corticosteroids do not prevent ARDS in at risk subjects [11-13]. Therapeutically, both high-dose and moderate-dose steroids have so far failed to demonstrate efficacy in ARDS. An ARDSnet randomized, double-blind trial in 180 patients with ARDS for more than seven days, showed no effect of prolonged treatment with moderate-dose methylprednisolone compared to placebo [14] (Table 1). Although patients were liberated from mechanical ventilation earlier, patients receiving methylprednisolone were more likely to resume assisted ventilation, which was thought to be secondary to neuromuscular effects. In addition, initiation of treatment after 14 days of ARDS was associated with a harmful effect, with increased mortality at 60 and 180 days.

However, the role of low-dose corticosteroids in established ARDS remains uncertain, with one study of 91 patients demonstrating prolonged low-dose methylprednisolone therapy reduces severity of lung injury by Day 7 of treatment [15] (Table 1).

Despite a systematic review [16] and meta-analysis [17], the role of steroids in ARDS remains unclear, and in light of ongoing uncertainty, further trials are both planned (NCT01731795) and on-going (NCT01284452) (Table 2). It is also worth highlighting that the studies included used what are now considered injurious ventilation strategies. It remains uncertain if steroids provide benefit when combined with lung protective (and, therefore, less inflammatory) ventilator strategies.

**Table 2:** Ongoing/planned clinical trials in ARDS

**Study reference number**	**Study title and abbreviation**	**Design**	**Population A) Timing B) P/F ratio**	**Anticipated enrollment**	**Intervention**	**Primary outcome**	**Status**
**NCT01731795**	Efficacy Study of Dexamethasone to Treat the Acute Respiratory Distress Syndrome (DEXA-ARDS)	Phase 2/3 RCT	A) 24 hours from ARDS onset B) <200	314	Dexamethasone (20 mg/day for five days, then 10 mg/day for five days)	Ventilator-free days	Not yet recruiting
**NCT01284452**	Efficacy of hydrocortisone in treatment of severe sepsis/septic shock patients with ALI/ARDS	Phase 2/3 RCT	A) 12 hours from organ dysfunction B) <300	194	Hydrocortisone 50 mg every six hours for seven days	28-day all-cause mortality	Recruiting
**ISRCTN88244364**	Simvastatin in acute lung injury (HARP-2) [20]	Phase 2/3 RCT	A) 48 hours from ALI onset B) <300	540	Simvastatin 80 mg daily	Ventilator-free days	Recruiting
**NCT00979121**	Statins for Acutely Injured Lungs from Sepsis (SAILS)	Phase 3 RCT	A) 48 hours from ALI onset B) <300	1000	Rosuvastatin 20 mg daily	Hospital mortality Day 60	Recruiting
**ACTRN12612000418875**	Nebulized heparin for lung injury	Phase 2 RCT	A) Within 24 hours of mechanical ventilation in at-risk patients B) <300	256	Nebulised Heparin 25,000 international units, every six hours for up to 10 days	Physical function assessed using physical function component of SF-36 health survey	Not yet recruiting
**NCT01659307**	The effect of Aspirin on REducing iNflammation in human *in vivo* model of Acute lung injury (ARENA)	Phase 2 RCT	Healthy, non-smoking adults, using an LPS model of ALI	33	Aspirin 75 mg or Aspirin 1,200 mg	Bronchalveolar lavage intraleukin-8 concentration	Not yet recruiting
**NCT01504867**	LIPS-A: Lung Injury Prevention Study with Aspirin	Phase 2 RCT	Adults admitted to hospital via the emergency department at high-risk of developing ALI	400	Aspirin 325 mg Day 1, then 81 mg daily days 2to 7	Development of ARDS	Recruiting
**ISRCTN95690673**	Keratinocyte growth factor in acute lung injury to reduce pulmonary dysfunction (KARE) [28]	Phase 2 RCT	A) 48 hours from ALI onset B) <300	60	Palifermin 60 μg/kg IV daily for up to six days	Oxygenation index at Day 7	Recruiting
**ISRCTN27673620**	VItamiN D Replacement to Prevent Acute Lung Injury following OesophagectOmy (VINDALOO) [34]	Phase 1/2 RCT	Adults undergoing planned transthoracic esophagectomy	80	Oral Vitamin D (100,000 IU)	EVLW at end of procedure	Recruiting
**NCT00789685**	Safety, tolerability and preliminary efficacy of FP-1201 in ALI and ARDS	Phase 1/2 Non-randomized	A) 48 hours from ALI onset B) <300	37	Interferon-β, increasing dose over six days	Clinically significant treatment emergent events, and all-cause mortality	Completed

### Statins

HMG CoA-reductase inhibitors (statins) have a range of physiological effects beyond their role in cholesterol reduction, including anti-inflammatory actions and endothelial function modulation. Their effect on pulmonary inflammation was confirmed during a randomized, double-blind, placebo-controlled pre-clinical study, where simvastatin demonstrated a variety of anti-inflammatory effects during an inhaled lipopolysaccharide (LPS) model of ARDS in healthy volunteers [18].

A small phase II clinical trial in patients with ARDS (HARP) [19], suggested a potential role for simvastatin in the treatment of ARDS, with benefit in pulmonary and non-pulmonary organ dysfunction with no excess of adverse events in the intervention group (Table 1). Two larger trials are presently recruiting in the UK and Ireland (HARP-2 [20]) and in the USA (SAILS, NCT00979121), investigating simvastatin and rosuvastatin, respectively. A phase two trial in Oklahoma was recently terminated due to poor enrollment (NCT01195428).

### Heparin

During the inflammatory process of ARDS fibrin is deposited throughout the alveolus, both intra- and extra-vascularly, impairing oxygenation. Experimental data show that, among other effects, heparin can reduce fibrin deposition. This led to a small study investigating the efficacy of nebulized heparin in patients at risk for ARDS [21]. Although there was no significant effect on the P/F ratio, this study suggested heparin may increase the number of ventilator-free days (VFD) (Table 1). The results of this trial have prompted further studies investigating the long-term impact of nebulized heparin in patients at risk of ARDS (ACTRN12612000418875) (Table 2).

### Aspirin

During ARDS, platelets become activated and play an important role in disease progression by sequestering within the lung, forming micro-thrombi and attracting inflammatory cells to injured tissue. The potent anti-platelet effect of aspirin may offer a therapeutic approach to this pathological process. Observational data associated pre-hospital anti-platelet use with a reduction in subsequent ARDS incidence [22]. This finding was repeated in a separate study, although when propensity to receive aspirin was included in the analysis, the effect was lost [23].

Clinical trials are planned to investigate the effect of aspirin on reducing inflammation in a human model of ARDS (ARENA, NCT01659307), while others are ongoing to assess the impact of aspirin in the prevention of ARDS (LIPS-A, NCT01504867) (Table 2).

### Angiotensin converting enzyme inhibitors/angiotensin receptor blockers

The renin-angiotensin system (RAS) plays an important role in the pathogenesis of ARDS, with angiotensin converting enzyme 1 (ACE1) directing a RAS signal to the angiotensin 1 receptor (AT1R), mediating alveolar vasoconstriction, permeability and fibrosis. A variant of ACE1, ACE2, diverts a RAS signal to AT2R, which promotes alveolar vasodilation, decreased permeability and apoptosis, thus opposing the alternative potentially injurious signaling mechanism [24]. Angiotensin receptor blockers attenuate ventilator-induced lung injury in animal models [25], and ACE-inhibitor or angiotensin receptor blocker therapy on discharge were associated with reduced mortality in acute respiratory failure patients [26]. Collectively, these data provide encouragement for future clinical trials in this area.

### Stem cell therapy

Regenerative medicine is an emerging field, using stem cells or growth factors to aid the repair of damaged tissue and organs. Stem cells exhibit anti-inflammatory, immunomodulatory and reparative effects, largely mediated through secreted growth factors, although cell to cell contact between stem cells and alveoli also mediates important effects [27].

This prompts questions regarding the optimal delivery of stem cell therapy, as animal models of ARDS have shown survival to increase when treatment was delivered directly to the bronchial tree [28]. In addition, recent evidence in *ex-vivo* human lung models of ALI support the investigation of delivering stem cells directly to the lung [29]. Clinical trials are awaited in this promising area.

### Growth factors

Keratinocyte growth factor (KGF), an epithelial growth factor secreted by fibroblasts, has an important role in lung injury repair [30]. It increases alveolar cellular proliferation in ARDS, particularly of type II alveolar cells, enhancing repair. KGF may also have a role during the injurious process, reducing endothelial permeability and alveolar edema [30], and improving alveolar fluid clearance [31]. Following the completion of a small pre-clinical trial testing KGF in an LPS-model of ARDS (ISRCTN98813895), for which results are awaited, a phase II trial has commenced investigating the efficacy and safety of intravenous KGF (palifermin) in ARDS [32] (ISRCTN95690673) (Table 2).

### Other potential therapies

Following data showing an immunomodulatory effect of vitamin D, animal models of ALI demonstrate that intra-tracheal administration of vitamin D can reduce neutrophil recruitment to the lung [33], which has obvious implications for future therapy in ALI. Vitamin D is currently being tested in patients at risk of developing ALI following esophagectomy [34] (Table 2).

Used in the management of multiple sclerosis, interferon-β (IFN-β) has been shown *in vitro* and in animal models of ALI to reduce vascular leakage and improve capillary endothelial barrier function [35]. IFN-β therapy has been studied in a phase I/II study in the UK, for which results are awaited (NCT00789685) (Table 2).

Finally, vascular endothelial growth factor (VEGF), an important molecule in the control of vascular permeability, has been found to be elevated in patients with ARDS [36]. The presence of VEGF inhibitors may prompt future randomized-controlled trials.

Other therapies, including the use of nitric oxide [37], prostacyclin [38] and surfactant [39], have been investigated and found to be ineffective. These additional therapies, plus others, are beyond the scope of this review, but have been covered in recent review articles [40,41]

## Conclusion

Despite many interventions being studied, to date there has been little success in developing effective pharmacological therapies for the management of ARDS. However, given the high associated morbidity and mortality, pressure remains to continue efforts to improve outcomes. Increasing numbers of pharmacological therapies are being investigated, and with encouraging pre-clinical and early clinical results, it is expected that over the coming years some will develop into useful agents for the prevention and treatment of ARDS.

## Abbreviations

ACE: Angiotensin converting enzyme; ALI: Acute lung injury; ALTA: Albuterol treatment for acute lung injury; ARDS: Acute respiratory distress syndrome; ARENA: The effect of Aspirin on REducing iNflammation in human *in vivo* model of Acute lung injury; AT1R: Angiotensin 1 receptor; AT2R: Angiotensin 2 receptor; BALTI: β-agonist Lung Injury Trial; CI: Confidence interval; DEXA-ARDS: Efficacy study of dexamethasone to treat the acute respiratory distress syndrome; EVLW: Extravascular lung water; HARP: A randomized clinical trial of hydroxymethylglutaryl–coenzyme a reductase inhibition for acute lung injury; IFN-β: Interferon-β; IV: Intravenous; KGF: Keratinocyte growth factor; LIPS-A: Lung injury prevention study with aspirin; LPS: Lipopolysaccharide; NE: Neutrophil elastase; NMB: Neuromuscular blockade; P/F ratio: PaO_2_/FiO_2_ ratio; RAS: Renin-angiotensin system; RR: Relative risk; SAILS: Statins for acutely injured lungs from sepsis; STRIVE: Neutrophil elastase inhibition in acute lung injury; VEGF: Vascular endothelial growth factor; VFD: Ventilator free days.

## Competing interests

DFM has performed paid consultancy work for GlaxoSmithKline relating to acute lung injury, is a member of the advisory boards for treatments of acute lung injury, and has been paid for undertaking bronchoscopy as part of a clinical trial. DFM has also received fees for lecturing for Astrazenica; and has a patent submitted for a novel treatment for ARDS. AJB and RMS have no competing interests to declare.

## Authors’ contributions

All authors contributed to the design of the paper. AJB wrote the initial manuscript, RMS and DFM edited it. All authors read and approved the final manuscript.

## Authors’ information

AJB is an Academic Foundation Year Two trainee. RMS is a specialist registrar in anesthesia and intensive care medicine. DFM is a professor and consultant in intensive care medicine. DFM has received funding from the Northern Ireland Public Health Agency Research and Development Division Translational Research Group for Critical Care. AJB and RMS are employed by the Belfast Health and Social Care Trust, while DFM has a joint appointment between Belfast Health and Social Care Trust and Queen’s University Belfast.

## Pre-publication history

The pre-publication history for this paper can be accessed here:

http://www.biomedcentral.com/1741-7015/11/166/prepub
